# The complete chloroplast genome sequence of *Michelia amoena* Q.F.Zheng et M.M.Lin

**DOI:** 10.1080/23802359.2021.2008821

**Published:** 2021-12-16

**Authors:** Qun Zhang, Site Luo, Yanqiu Xie, Linting Zhang, Chuanyuan Deng

**Affiliations:** a College of Landscape Architecture, Fujian Agriculture and Forestry University, Fuzhou, Fujian, China; b School of Life Sciences, Xiamen University, Xiamen, China; c Island Research Center, Ministry of Natural Resources, Pingtan, Fujian, China

**Keywords:** *Michelia amoena*, chloroplast genome, phylogeny

## Abstract

*Michelia amoena* Q.F.Zheng et M.M.Lin is classified in Magnoliaceae and has a high economic value. Herein, we report the complete chloroplast genome of *M. amoena* using Illumina sequence data. The chloroplast genome is 160,088 bp in length and contains a large single copy (LSC) region of 88,118 bp and a small single-copy (SSC) region of 18,798 bp separated by two inverted repeat (IR) regions of 26,586 bp each. It contained a total of 131 genes, with an overall GC content of 39.26%. The phylogenetic analysis showed that *M. amoena* is closely related to *Michelia figo*. This study provides important sequence information for species identification and its phylogenetic relationship in the Magnoliaceae.

*Michelia amoena* Q.F.zheng et M.M.lin (*Michelia*, Magnoliaceae) was found in the forest at an altitude of 700 meters of Changting, Fujian Province, China and first reported as a new species in 1987 in the journal *Bulletin of Botanical Research* (Zheng and Lin 1987). Recently, *Michelia amoena* has been merged into *Michelia skinneriana* and treated as a synonym of *Michelia skinneriana* in the revised monograph *China of Flora* since two species share great similarities in morphological features. However, in our field invstigation at type locality of *Michelia amoena* for local germplasm resources of woody plants, we found *Michelia amoena* and *Michelia skinneriana* can co-occur in the same forest as understory shrubs and easily be recognized in that the former bears obovate or obovate-elliptic leaf blade, short and acute leaf apex, and stipule scars extending to the middle of the petiole besides lavender and glabrous tepals which can only be observed in blooming periods, while the latter bears oblanceolate leaf blade, long caudate-acuminate leaf apex, and stipular scar extending to petiole apex besides white and brown tomentose at tepal base which can only be observed in blooming periods, which make the decision to merge *Michelia amoena* into *Michelia skinneriana* unstable. Therefore, we collected the *Michelia amoena* samples at type locality for first cp DNA analysis, which might provide basic molecular evidence for species circumscription. Here we performed high-throughput sequencing on a specimen of *M. amoena* from China to determine its cp genome structure and evolutionary relationship to Magnoliaceae.

The classification of Magnoliaceae is controversial, Many genera including *Michelia* had recently been merged with *Magnolia* . However, in the monograph ‘Flora of China,’ the genera *Michelia* and *Magnolia* are two seperate genera. We adopt the systematic classification of Magnoliaceae accepted by the monograph ‘Flora of China’ in this article, which is different from that to recognize only the genus *Magnolia* in family Magnoliaceae. The following checklist is provided for the benefit of those who prefer to recognize Magnoliaceae to include only the genus *Magnolia.* All the names accepted in the present treatment (in italics) are cross-referenced to the corresponding names in *Magnolia* (in boldface) (Xia et al. 2008).

*Michelia figo* = Magnolia figo
*Michelia yunnanensis* = Magnolia laevifolia
*Michelia maudiae* = Magnolia maudiae
*Michelia chapensis* = Magnolia chapensis
*Michelia alba* = Magnolia alba
*Michelia compressa* = Magnolia compressa
*Manglietia crassipes* = Magnolia crassipes
*Michelia skinneriana =* Magnolia figo var. skinneriana

The fresh leaves of *M. amoena* were collected from dense forests in Changting County, Longyan, Fujian Province, China (25°32′23″N, 116°4′49″E). The voucher specimen is deposited at Fujian Agriculture and Forestry University (No. FZ-FJ2021-01A, FAFU, QunZHANG: zq225512@gmail.com). The genomic DNA was extracted using Plant Genomic DNA Kit, DP305 (TIANGEN, Beijing, China). The sequencing library was produced using the Illumina Trueseq™ DNA Sample Preparation Kit (Illumina, San Diego, USA) according to the manufacturer's recommendations. The prepared library was loaded on the Illumina Novaseq 6000 platform for PE 2 × 150 bp sequencing at Novogene (Beijing, China). The raw data were used to assemble the complete cp genome using the GetOrganelle pipeline (Jin et al. 2020). Genome annotation was performed with PGA (Qu et al. 2019) by comparing the sequences with the cp genome of *Michelia figo*, GenBank Accession Number NC_053861(Zhai 2020). The annotated genome sequence was deposited in GenBank under Accession Number MZ297474.

The circular cp genome of *M. amoena* was 160,088 bp and contains a larger single copy (LSC) region of 88,118 bp in length, a smaller single copy (SSC) region of 18,798 bp in length, and a pair of inverted repeats (IR) of 26,586 bp. There were 131 genes predicted in this genome, of which 86 are protein-coding genes, 37 tRNA and 8 ribosomal RNA genes. The base content of the *M. amoena* cp genome is A (29.98%), T (30.76%), C (19.98%), G (19.28%) and the overall GC content of the cp genome is 39.26%.

The cp genome of *M. amoena* was aligned with other 9 cp genomes of Magnoliaceae and *Liriodendron chinense* were designated as the outgroup to construct the phylogenetic tree. All complete cp genomes were aligned with the MAFFT v7.388 using default settings (Katoh and Standley 2013). The phylogenetic analysis was conducted based on maximum likelihood (ML) analyses implemented in IQ-TREE v2.1.2 with the TVM + F+R5 nucleotide substitution model, which was selected by ModelFinder (Kalyaanamoorthy et al. 2017; Minh et al. 2020). The support for the inferred ML tree was inferred by bootstrapping with 1,000 replicates. The phylogenetic analysis suggested that *M. amoena* was closely related to *M. figo* (Figure 1). This chloroplast genome will provide an important resource for addressing taxonomic issues and studying the molecular evolution of Magnoliaceae.

**Figure 1.**
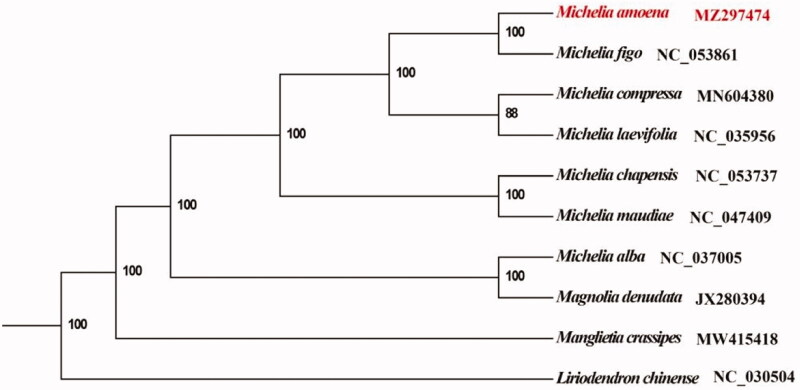
Maximum-likelihood (ML) tree based on 9 complete cp genome sequences from the Magnoliaceae with *Liriodendron chinensis* designated as outgroup. Numbers on the nodes are bootstrap values based on 1,000 replicates. *Michelia amoena* was marked in red.

## Data Availability

The genome sequence data that support the findings of this study are openly available in GenBank of NCBI at (https://www.ncbi.nlm.nih.gov/) under the accession no MZ297474. The associated BioProject, SRA, and Bio-Sample numbers are PRJNA732387, SAMN19317494, and SRR14660103, respectively.
